# Pyogenic Hepatic Abscess: A Case Report and Literature Review on a Rare Complication of Gastric Sleeve Surgery

**DOI:** 10.7759/cureus.22650

**Published:** 2022-02-27

**Authors:** Deema Chakhachiro, Abdul Rahman Al Armashi, Ameed Bawwab, Isaac Alsallamin, Mohamed Homeida, Dina Haroun, Mohammad Haidous, Keyvan Ravakhah

**Affiliations:** 1 Department of Internal Medicine, Northeast Ohio Medical University, Cleveland, USA; 2 Department of Internal Medicine, Saint Vincent Charity Medical Center, Cleveland, USA

**Keywords:** review of literature, gastric maltoma, rare complication, gastric sleeve surgery, pyogenic hepatic abscess

## Abstract

Gastric sleeve surgery is a frequently performed procedure. Although it is one of the safest bariatric procedures, it is like any other operation that carries significant risks and complications. Moreover, the hepatic abscess is an infrequent complication of laparoscopic gastric sleeve surgery, the infected late gastric leakage is a rare etiology of the hepatic abscess. We present a case of liver abscess developed one month after sleeve gastrectomy secondary to infected walled-off late-gastric leak. The case highlights this rare complication of gastric sleeve surgery. Moreover, early treatment of liver abscesses with imaging-guided drainage and intravenous antibiotics can prevent life-threatening outcomes.

## Introduction

Gastric sleeve surgery is a frequently performed procedure to aid individuals with severe obesity in losing weight. It is typically performed laparoscopically and about 80% of the stomach is removed leaving only a small part [1]. Although it is one of the safest bariatric procedures, like any other operation, carries significant risks and complications such as gastrointestinal obstruction, hernias, gastroesophageal reflux and rarely can be fatal [1]. The gastric leak is considered the most serious complication since it is associated with a high mortality rate [2]. Gastric leaks can be due to mechanical or ischemic causes. The mechanical causes are due to intraoperative complications, such as misfiring or direct tissue injury, which usually appears within two days of surgery. While the ischemic causes appear on days 5-6 postoperatively [3]. Interestingly, gastric sleeve surgery has the longest staple line, increasing the risk of leakage [4]. The infected leakage may cause a hepatic abscess or splenic abscess, which has been rarely reported in the literature in gastric sleeve surgery patients. We report a case of a 64-year-old female who developed a pyogenic hepatic abscess one month after gastric sleeve surgery.

## Case presentation

Our patient is a 64-year-old woman with a past medical history significant for hypertension and morbid obesity (BMI 40 kg/m^2^) who underwent a laparoscopic sleeve gastrectomy one month before her current presentation. She presented to the emergency department with progressive, sharp, localized epigastric pain for two days. The pain had no alleviating or aggravating factors and did not have radiation. The review of systems was significant for nausea, anorexia, and subjective fever. Physical exam was significant for fever (40^o^C), clear lung fields, and normal heart sounds. Her abdominal examination was remarkable for severe right upper quadrant tenderness with negative peritoneal signs. Laboratory work showed leukocytosis; otherwise, electrolytes, blood urea nitrogen, aminotransferases, alkaline phosphatase, bilirubin, lipase, and amylase were within normal limits (Table 1).

**Table 1:** Laboratory results BUN: blood urea oxygen; GFR: glomerular filtration rate; AST: aspartate aminotransferase; ALT: alanine transaminase

Variables	Value	Reference range
WBC	13.4 K/Ul	3.9-11
Na	134 mmol/L	136-145
K	3.7 mmol/L	3.5-5.1
Cl	99 mmol/L	98-107
Carbon dioxide	25 mEq/L	23-29
Creatinine	0.79 mg/dl	0.7-1.3
BUN	18 mg/dl	7-18
GFR	>60	>60
Total bilirubin	0.5 mg/dl	1-1.2
AST	29 U/L	5 - 40
ALT	32 U/L	7 - 56
Alkaline phosphatase	115 IU/L	44 - 147
Amylase	100 U/L	25-115
Lipase	60 U/L	0-160

**Figure 1:**
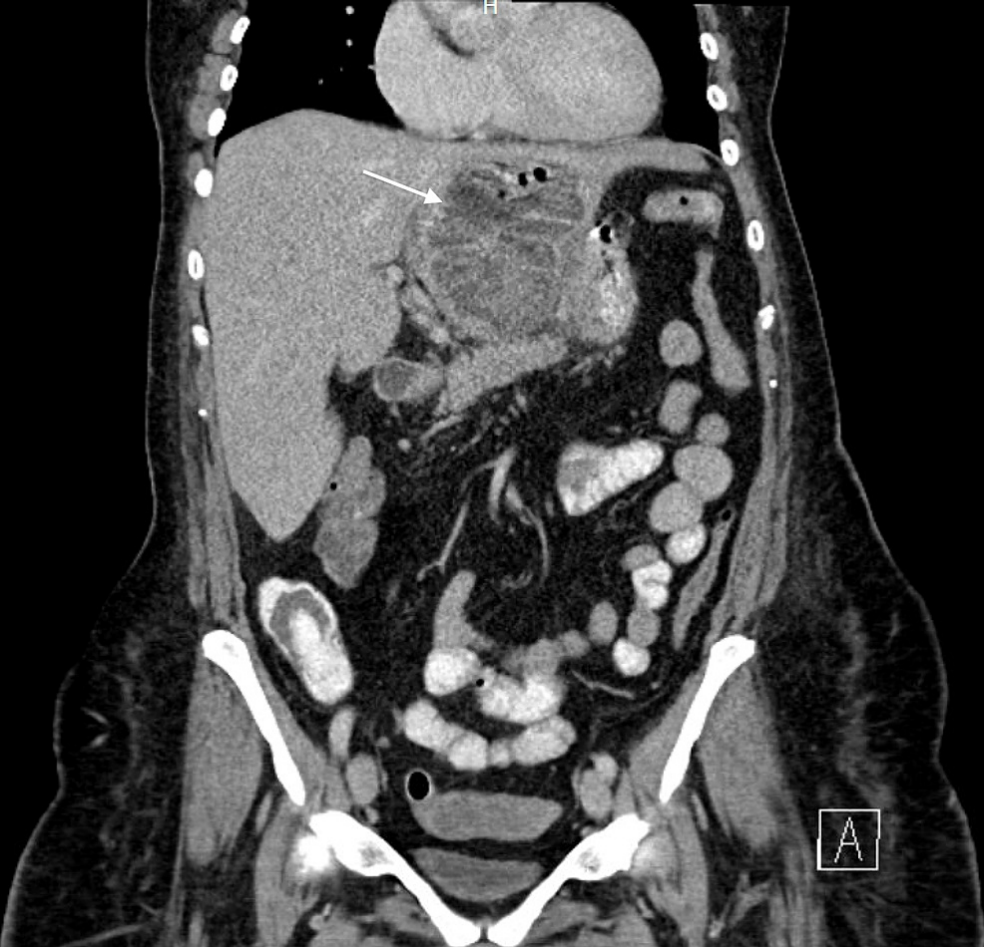
CT scan of the abdomen with intravenous contrast shows a liver abscess of the left lobe with multiple fluid levels on the coronal plane (white arrow)

**Figure 2:**
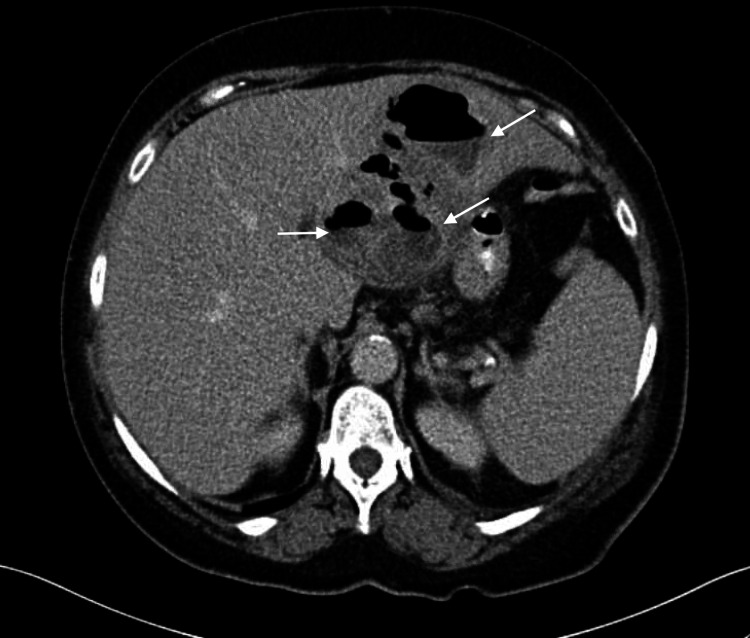
CT scan of the abdomen with intravenous contrast shows liver abscess of the left lobe with multiple fluid levels on the sagittal plane (white arrow)

The upper gastrointestinal series was negative for an acute leak. Subsequently, a computed tomography (CT) scan of the abdomen with intravenous (IV) contrast was performed and revealed a liver abscess of the left lobe with multiple air/fluid levels on the coronal and sagittal plane, respectively (Figures 1-2).

She was managed with intravenous (IV) antibiotics and pain management. A few hours later, the patient's symptoms continued to progress with worsening fever and tachycardia requiring a transfer to the intensive care unit, and urgent CT-guided drainage of the liver abscess was successfully performed. The patient was discharged following stabilization on oral antibiotics. Furthermore, a follow-up CT scan of the abdomen showed resolution of the abscess.

## Discussion

Laparoscopic sleeve gastrectomy is rapidly gaining popularity as a bariatric procedure for obese individuals. While this procedure has several advantages, serious complications, such as staple line bleeding, leaks, and gastric strictures, are possible [2].

The hepatic abscess is an infrequent complication after laparoscopic sleeve gastrectomy. Many etiologies play a role in the formation of liver abscesses, such as enteric fistula formation, the spread of the bacteria from the infected material in the gastrosplenic area after laparoscopic sleeve gastrectomy, and ascending migration of bacteria from the portal venous system due to pylephlebitis of the portomesenteric veins secondary to leak-related collection [4].

In this case, the etiology of the liver abscess is unknown. The upper gastrointestinal series did not show enteric fistula, and the CT scan of the abdomen did not show pylephlebitis. A leak can be classified according to the time of its emergence, as an early leak that may appear between days 1 and 3 after surgery, an intermediate leak between days 4 and 7 after surgery, and a late leak that develops during or after day 8 of surgery [5].

Gastrografin upper gastrointestinal (GI) series examinations are helpful to establish leaks at the gastro-jejunostomy or upper gastric pouch staple line after laparoscopic gastric bypass surgery. However, they do not definitively rule out leaks in other locations, which explains the false-negative findings of the upper gastrointestinal series in this patient [6]. For this reason, any patient with tachycardia, fever, and leukocytosis after laparoscopic sleeve gastrectomy warrants extensive exploration.

Furthermore, the liver abscess could be fatal due to severe complications such as sepsis, empyema, and peritonitis. CT or US-guided percutaneous needle aspiration with or without drainage catheter is the first management choice. Laparoscopic or open surgical drainage will be the next step if the percutaneous aspiration is inadequate [7]. Moreover, catheter placement is required when the abscess is greater than 5 cm. The aspirate should be sent for gram stain and aerobic/anaerobic culture. Besides the abscess drainage, patients should be on empiric coverage for Enterobacteriaceae, Enterococci, anaerobes, Staphylococci, and Streptococci in certain situations. Depending on the culture results, these antibiotics may be narrowed later [8]. Antibiotics as a sole treatment are the best choice for patients who do not tolerate invasive surgery or patients with non-amenable multiple abscesses [8]. In the presence of a staple line leak, endoscopic interventions, including covered stent placement or clip usage, are options to close the defective area [9]. Active leakage was ruled out in this case, and management with CT-guided percutaneous drainage and antibiotics was sufficient.

Upon literature review, five cases of liver abscess after laparoscopic sleeve gastrectomy were found. The male to female ratio was equal. The time frame of abscess formation post-surgery ranged from two weeks to six months. Furthermore, our patient was similar to the five patients, where complete recovery was achieved with antibiotics administration and the drainage of the abscess (Table 2).

**Table 2:** Literature review of hepatic abscess after gastric sleeve surgery

Case	Age	Gender	Time from Surgery to abscess	EGD finding	Treatment	Outcome
Alfalah et al. [10]	32	female	6 weeks	No leakage	antibiotic and drainage	Recovered
Demir H et al. [4]	37	female	2 weeks	No extra-luminal contrast extravasation.	antibiotic and drainage	Recovered
Al Faris H et al. [11]	37	male	10 days	Very small opening measuring 4 mm at the gastroesophageal junction	antibiotic and drainage	Recovered
Abdelhady MH et al. [9]	42	female	6 months	Very small leak opening	antibiotic and drainage	Recovered
Abdelhady MH et al. [9]	32	male	6 months	No leakage	antibiotic and drainage	Recovered

## Conclusions

This case highlights a rare complication of gastric sleeve surgery. It also illustrates that the upper gastrointestinal series is not a definitive diagnostic test of leakage. A CT scan of the abdomen with IV and oral contrast is advised, especially when a patient presents with fever, tachycardia, and abdominal pain. Moreover, early treatment of liver abscess with imaging-guided drainage and intravenous antibiotics has a favorable outcome.
